# Relative Vaccine Effectiveness of Adjuvanted Trivalent Influenza Vaccine over Three Consecutive Influenza Seasons in the United States

**DOI:** 10.3390/vaccines10091456

**Published:** 2022-09-02

**Authors:** Constantina Boikos, Ian McGovern, Justin R. Ortiz, Joan Puig-Barberà, Eve Versage, Mendel Haag

**Affiliations:** 1 Seqirus Inc., Kirkland, QC H9H 4M7, Canada; 2 Seqirus USA, Cambridge, MA 02139, USA; 3 Center for Vaccine Development and Global Health, University of Maryland School of Medicine, Baltimore, MD 21201, USA; 4 Foundation for the Promotion of Health and Biomedical Research (FISABIO), 46020 Valencia, Spain; 5 Seqirus Inc., 1101 CL Amsterdam, The Netherlands

**Keywords:** influenza, influenza vaccines, relative vaccine effectiveness, immunosenescence

## Abstract

Traditional influenza vaccines may be less immunogenic in adults ≥65 years of age due to immunosenescence. Two influenza vaccines—MF59®-adjuvanted trivalent inactivated influenza vaccine (aIIV3) and high-dose influenza vaccine (HD-IIV3)—were developed to overcome this problem. We summarize estimates of the relative vaccine effectiveness (rVE) of aIIV3 vs. HD-IIV3 and aIIV3 vs. standard, egg-based quadrivalent influenza vaccines (IIV4e) during the 2017–2018, 2018–2019, and 2019–2020 US influenza seasons using the same underlying electronic medical record and linked claims dataset for all three seasons. The primary outcome was influenza-related medical encounters (IRMEs), defined by diagnostic codes specific to influenza (ICD J09*-J11*). rVE was estimated using propensity score methods adjusting for demographics and health status. rVE estimates demonstrated consistent benefit for aIIV3 over IIV4e in the overall and at-risk populations. Relative to HD-IIV3, aIIV3 provided improved benefit in the overall study population and comparable benefit in the at-risk population across each season.

## 1. Introduction

Real-world evidence (RWE) represents an important component of evaluation of seasonal influenza vaccine performance. Randomized controlled trials (RCTs) provide essential information on vaccine efficacy and safety. RCTs are conducted during a single or limited number of influenza seasons and generally in healthy study subjects. Influenza vaccine effectiveness determined from RWE can complement RCTs to assess vaccine performance. RWE enables evaluation of the effectiveness of vaccines in larger and more inclusive populations over longer time periods [1,2].

Public health agencies, such as the US Centers for Disease Control and Prevention (CDC) and the World Health Organization (WHO), use RWE to monitor vaccine performance [3,4,5,6,7]. This information is used to inform influenza vaccine policy, vaccine strain selection, clinical practice guidelines, health communications, and product development [6,7,8,9,10,11,12,13]. 

In influenza vaccine research, RWE can be generated from a wide variety of data sources, including medical and pharmacy insurance claims and medical records [14]. Such datasets enable the estimation of how well different vaccines work and their effectiveness against various outcomes under different circumstances of seasonal characteristics. The volume and variety of the information permits assessment of relative vaccine effectiveness (rVE) within and between subgroups (based on age or health status), and can provide insights into the impact of vaccination on public health, with large study samples supporting the estimation of more precise effect estimates.

Age-related decreases in immune responses—known as immunosenescence—frequently leave older adults vulnerable to influenza and its complications [15,16]. In the US, approximately 90% of influenza-related deaths, including those due to secondary pneumonia and occurring among people with pre-existing cardiovascular and respiratory diseases, are recorded in persons aged ≥65 years [17,18,19,20]. Two enhanced influenza vaccines—MF59®-adjuvanted trivalent inactivated influenza vaccine (aIIV3) and high-dose influenza vaccine (HD-IIV3)—were specifically developed for use in older adults to overcome the challenge of immunosenescence.

The effectiveness of adjuvanted trivalent inactivated influenza vaccine (aIIV3) has been reviewed in a previous systematic review and meta-analysis [21]. Differences in the underlying study population can introduce variability into evaluation of influenza vaccine effectiveness, complicating the evaluation of vaccine performance obtained from a variety of data sources. Here, we focus on a specific subset of the published literature on the effectiveness of aIIV3 that used the same data source over three consecutive influenza seasons from 2017 through 2020 to describe the effectiveness of aIIV3 relative to egg-based quadrivalent inactivated influenza vaccine (IIV4e) and high-dose trivalent inactivated influenza vaccine (HD-IIV3) in the United States [22,23,24]. The focus on studies that utilized the same methodologies and databases across each season allows for better evaluation of how the differences in the underlying epidemiological characteristics of the influenza seasons may have contributed to variation in the rVE of aIIV3 vs. IIV4e and HD-IIV3. The data source contains electronic medical records and claims data from 3.4 to 5.8 million vaccinated subjects per season who were ≥65 years of age and received aIIV3, HD-IIV3, or IIV4e. Each study evaluated the rVE of aIIV3 vs. each of the other two vaccines against outcomes defined by diagnostic codes for influenza-related medical encounters (IRME). Additionally, we review the influenza epidemiological characteristics during the season under study to understand how this may have affected the relative effectiveness of aIIV3 compared to HD-IIV3 and IIV4e.

## 2. Methodology Used to Determine Estimates of Relative Vaccine Effectiveness

Three retrospective cohort studies were conducted during the 2017–2018, 2018–2019, and 2019–2020 influenza seasons in individuals ≥65 years of age in the United States [22,23,24]. All three studies used the same integrated dataset comprising de-identified (anonymous) data from electronic medical records (EMRs) from primary care and specialty clinics (Veradigm Health Insights Ambulatory database; Allscripts Touchworks and Allscripts PRO, Chicago, IL, and Practice Fusion, Inc., San Francisco, CA, USA). These EMRs were also linked to additional pharmacy and medical claims where available (Komodo Healthcare Map, Komodo Health Inc., New York, NY, USA). All datasets included only de-identified clinical data that met Protected Health Information data requirements and were certified for Health Insurance Portability and Accountability Act (HIPAA) compliance [22,23,24]. The studies were conducted and the findings were reported in accordance with Good Pharmacoepidemiological Practice, the Declaration of Helsinki, applicable local regulations, and the Reporting of Studies Conducted using Observational Routinely Collected Health Data (RECORD) [25]. 

The study populations consisted of individuals ≥65 years of age who had a record of vaccination with aIIV3, IIV4, or HD-IIV3 during each season (Table S1). Subjects were vaccinated between August 1 and February 28 of the 2017–2018 and 2018–2019 seasons, and the observation period spanned the full influenza season (1 October 2017, through 19 May 2018, and 30 September 2018, through 18 May 2019) [22,26,27]. During the 2019–2020 season, eligible subjects were vaccinated between 1 August and 31 January, and the observation period ended on 7 March 2020, to avoid overlap with circulation of SARS-CoV-2 in the US [24]. The primary outcome during each season was occurrence of IRMEs defined using International Classification of Diseases (ICD)–9-CM and ICD-10-CM codes specific to the diagnosis of influenza disease (Table S2) [28].

In all three seasons, adjusted rVE estimates were derived using a doubly robust inverse probability of treatment weighted (IPTW) approach. First, propensity scores were calculated for each subject using a logit model predictive of treatment group membership, and then the propensity scores were used to create stabilized weights [29]. For analyses of the 2017–2018 and 2018–2019 seasons, variables included age, sex, race, ethnicity, geographic region, vaccination week, and health status. For the analysis of the 2019–2020 season, three additional variables were adjusted for, in addition to those from previous seasons: frailty (proxied by an index for activities of daily living) [30], Charlson comorbidity index (CCI) [31,32], and the number of outpatient visits and inpatient admissions in the year before vaccination [33]. For all three seasons, a doubly robust adjustment methodology was used to estimate adjusted odds ratios (OR_adjusted_) for the overall population and subgroups (age and high risk), and adjusted rVE was determined using the formula (% VE = 1 − OR_adjusted_) × 100. Sensitivity analyses were conducted to evaluate rVE during peak influenza activity. Peak influenza activity period was defined based on an analysis of data from the US CDC on the percent of outpatient influenza tests that were positive for influenza using the Moving Epidemic Method [34,35]. Additional details on the study methodologies, including information on changes to inclusion/exclusion criteria, can be found in the original publications [22,23,24].

## 3. Seasonal Characteristics and Relative Effectiveness of aIIV3 between 2017 and 2020

### 3.1. Burden of Influenza and Overall Vaccine Effectiveness

**Figure 1:**
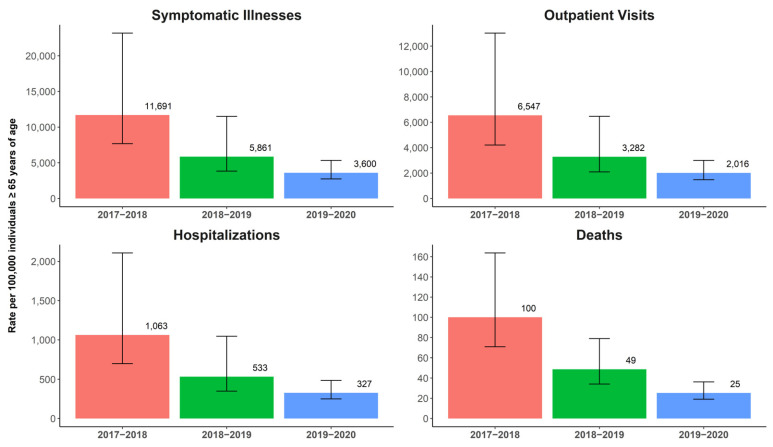
Estimated incidence rates (and 95% uncertainty interval) of influenza-related outcomes (symptomatic illnesses, outpatient visits, hospitalizations, and deaths) per 100,000 individuals ≥65 years of age as estimated by the US Centers for Disease Control and Prevention (CDC) for seasons from 2017–2018, 2018–2019, and 2019–2020 [36,38,39].

**Figure 2:**
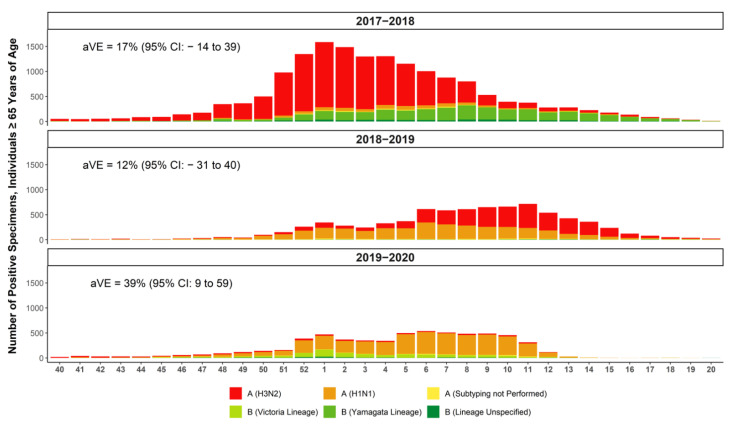
Summary of influenza-positive specimens, specified by strain, from individuals ≥65 years of age, as reported by public health laboratories across the United States to the CDC and absolute vaccine effectiveness (aVE) against any strain as estimated by the CDC for individuals ≥65 years of age [26,27,37,40,41,42].

Figure 1 summarizes the CDC-estimated morbidity and mortality of influenza among adults ≥65 years of age, and Figure 2 presents the predominant circulating strains and absolute vaccine effectiveness among adults age ≥65 years of age during each season in the US. The 2017–2018 season was a high severity season dominated by the A(H3N2) strain, with some B/Yamagata circulation [26,36]. The US CDC estimated the absolute vaccine effectiveness for any type of vaccine that year to be 17% (95% CI, −14% to 39%) in adults ≥65 years of age [37]. The 2018–2019 and 2019–2020 seasons had decreased severity compared to the 2017–2018 season, with lower rates of infections and hospitalizations [27,38,39]. Predominant circulating strains during the 2018–2019 season were split between A(H1N1), which was most abundant from October 2018 to mid-February 2019, and A(H3N2), which dominated from February through May 2019. The US CDC estimated that the overall vaccine effectiveness among ≥65 years was 12% (95% CI, −31% to 40%) [27]. In the 2019–2020 season, the predominant strain in adults ≥65 years of age was A(H1N1), and the absolute vaccine effectiveness was 39% (95% CI, 9% to 59%) in those ≥65 years of age [40,41,42].

### 3.2. Overall Relative Vaccine Effectiveness of aIIV3 vs. HD-IIV3 and IIV4e

Table 1 summarizes the numbers of subjects in each vaccine group in each season. The most common demographic characteristics of the study populations (individual characteristic with a frequency of >50%) during all three seasons were ethnicity/race of non-Hispanic/white, geographic residence in the South, and female sex. Demographic differences between vaccine groups were minimal after weighting, as described in the original publications [22,24]. The relative distribution of vaccines by type among high-risk individuals was similar to the distributions in the overall populations for the 2017–2018 and 2018–2019 seasons.

**Figure 3:**
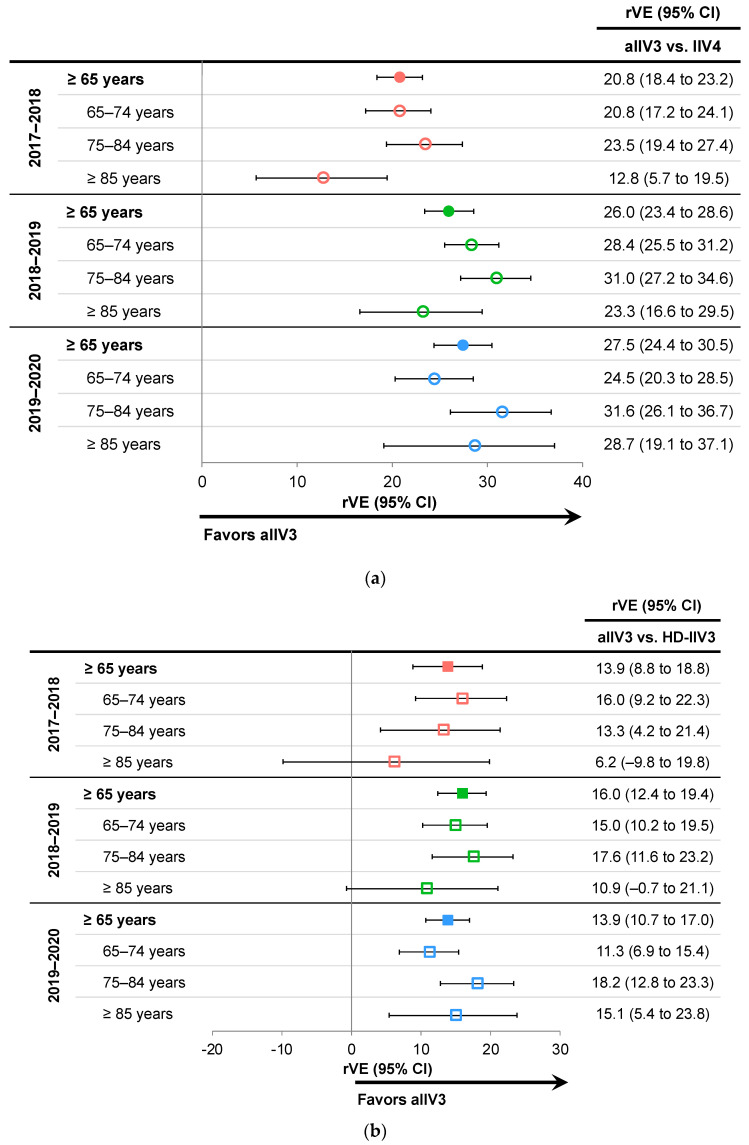
Relative vaccine effectiveness (rVE) of adjuvanted trivalent inactivated influenza vaccine (aIIV3) vs. comparators for the prevention of influenza related medical encounters during the three influenza seasons between 2017 and 2020 in subjects ≥65 years of age and by age subgroups [22,24]. (**a**) aIIV3 vs. egg-based quadrivalent inactivated influenza vaccine (IIV4e); (**b**) rVE for aIIV3 vs. high-dose trivalent inactivated influenza vaccine (HD-IIV3). CI, confidence interval.

The overall rVE favored aIIV3 over IIV4e for the prevention of influenza related medical encounters with estimates ranging between 20.8% (95%CI: 18.4 to 23.2) and 27.5% (95%CI: 24.4 to 30.5). Similar effect sizes were observed in the subgroups of age even among those ≥85 years, with the exception of the 2017–18 season (12.8%; 95%CI: 5.7 to 19.5) (Figure 3a) [22,24]. For the comparison of aIIV3 vs. HD-IIV3 during all three seasons, rVE estimates favored aIIV3 vs. HD-IIV3 in the overall populations, with estimates ranging from 13.9% (95%CI: 8.8 to 18.8) to 16.0% (95%CI: 12.4 to 19.4) (Figure 3b) [22,24]. When analyzed by age subgroups (65–74, 75–84, and ≥85 years), point estimates for the aIIV3 vs. HD-IIV3 comparison favored aIIV3, except in the ≥85-year subgroup in 2017–2018 and 2018–2019, for which comparable performance of both vaccines was observed [22]. The relative benefit of aIIV3 vs. IIV4e (overall rVE point estimate range: 20.8% to 27.5%) was larger than relative to HD-IIV3 (overall rVE point estimate range: 13.9% to 16.0%).

### 3.3. rVE during Peak Influenza Activity

**Figure 4:**
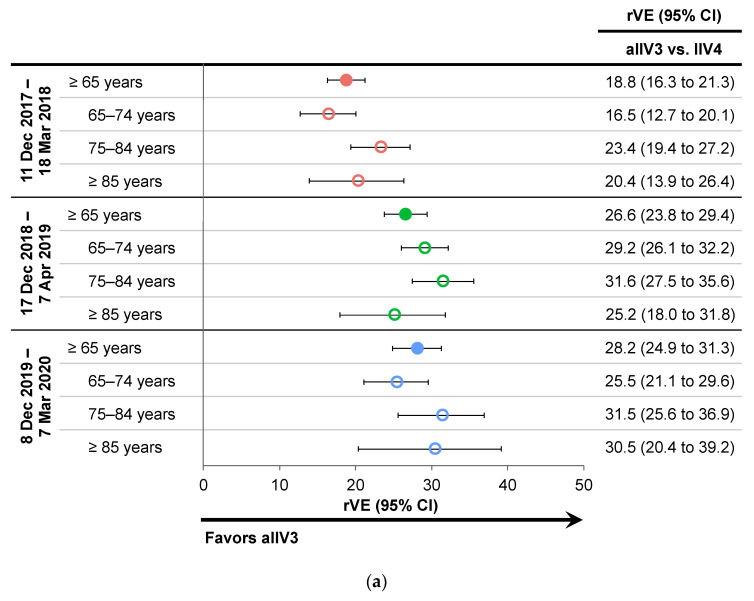

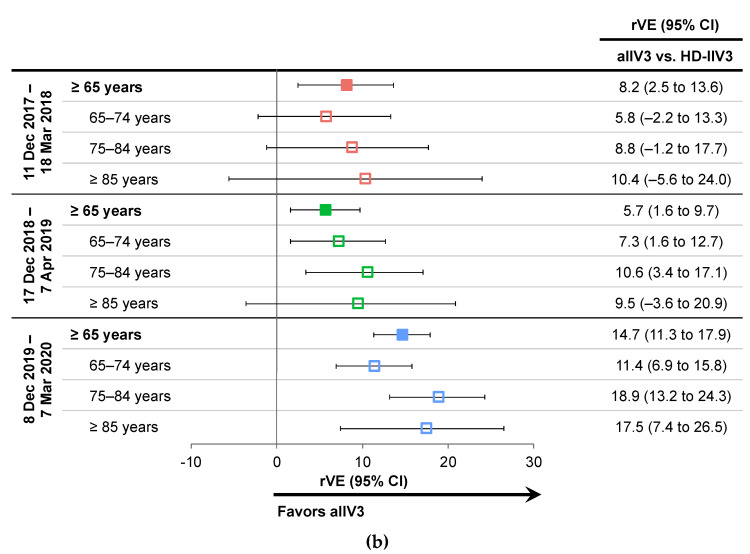
Relative vaccine effectiveness (rVE) of adjuvanted trivalent inactivated influenza vaccine (aIIV3) vs. comparators during periods of peak influenza activity between 2017 and 2020 in subjects ≥65 years of age and by age subgroups [22,24]. (**a**) aIIV3 vs. egg-based quadrivalent inactivated influenza vaccine (IIV4e); (**b**) rVE for aIIV3 vs. high-dose trivalent inactivated influenza vaccine (HD-IIV3). CI, confidence interval.

Between 2017 and 2020, the periods with the highest laboratory-confirmed influenza activity was estimated using a moving epidemic method to have occurred between 11 December 2017, and 18 March 2018; 17 December 2018, and 7 April 2019; and 8 December 2019, and 7 March 2020 (Figure 2) [34,35]. Similar to the primary analysis, aIIV3 was significantly more effective than IIV4e in all age subgroups during the peak influenza periods of all three seasons, with similar rVE point estimates in both the primary and peak season analysis (Figure 4a) [22,24]. rVE during these periods of peak activity suggested a smaller but still significant advantage of aIIV3 vs. HD-IIV3 in the overall population during the 2017–2018 and 2018–2019 seasons, and a slightly greater advantage in the 2019–2020 season compared to the main analyses (Figure 4b) [22,24].

### 3.4. rVE in Subjects at High Risk of Influenza Complications

During the 2017–2018 and 2018–2019 seasons, additional analyses examined the prevention of IRMEs in nearly four million individuals, with at least one medical condition putting them at high risk of influenza complications. Note that this category is separate from an age above 65 years, which itself constitutes a risk factor for influenza as per the US CDC. High-risk conditions included chronic pulmonary diseases, heart disease (myocardial infarction or congestive heart failure), cerebrovascular disease, renal disease, diabetes, malignancy or metastatic solid tumors, HIV/AIDS, rheumatic disease, and liver disease [23,43]. In the overall high-risk population, aIIV3 and HD-IIV3 provided similar protection from IRMEs, with an overall rVE of −0.8% (95% CI, −8.9% to 6.6%) in 2017–2018 and 2.7% (−2.7% to 7.8%) in 2018–2019. In the comparison between aIIV3 and IIV4e among high-risk individuals, the overall rVE was 7.1% (3.3% to 10.8%) in 2017–2018 and 20.4% (16.2% to 24.4%) in 2018–2019 [23]. 

### 3.5. Outpatient and Inpatient Visits 

In the 2019–2020 season, an exploratory (per-protocol) analysis evaluated IRMEs separately for outpatient and inpatient medical settings. aIIV3 demonstrated greater protection against outpatient visits for influenza compared to HD-IIV3 (16.9% (95% CI, 13.2% to 20.4%)) or IIV4e (31.3% (27.8% to 34.6%)). Inpatient IRMEs were also reduced with aIIV3, with rVEs of 6.5% (0.1% to 12.4%) vs. HD-IIV3 and 17.1% (10.8% to 23.2%) vs. IIV4e.

## 4. Discussion

In adults ≥65 years of age, comparisons between aIIV3 and HD-IIV3 and between aIIV3 and IIV4e consistently favor aIIV3 across three influenza seasons between 2017 and 2020. The benefit was larger in the comparison of aIIV3 vs. IIV4e than aIIV3 vs. HD-IIV3, which is in line with expectation and supports the use of adjuvanted over standard-dose, non-adjuvanted influenza vaccines for older adults. Variability in rVE is expected depending on the epidemiological characteristics of a specific influenza season and the populations being studied. For example, during the 2017–2018 season, approximately 24% of circulating viruses among adults ≥65 years of age were B(Yamagata). The trivalent formulation of influenza vaccines contained B(Victoria) and not B(Yamagata) virus for all three seasons; as such, B(Yamagata) infections in the 2017–2018 season were not addressed by the trivalent formulation of the influenza vaccine [44]. Even so, we hypothesize that the rVE estimate from this season suggests that the increased magnitude and breadth of immune response conferred by the MF59® adjuvant in a trivalent formulation may have outweighed the advantage offered by the fourth strain in a standard-dose, non-adjuvanted quadrivalent vaccine. A quadrivalent formulation of the MF59® adjuvanted vaccine was later approved in 2020 in the US (and first available in the 2020–2021 season). The greatest benefit of the aIIV3 compared to HD-IIV3 was observed in the 2018–2019 season, when an antigenically drifted form of A(H3N2) was circulating; in this case, inclusion of an adjuvant would be expected to confer a benefit, since the higher dose of antigen is not expected to improve effectiveness against the drifted strain [34,45].

When considering the low absolute vaccine effectiveness during the first two seasons (17% in 2017–2018 and 12% in 2018–2019), the 20.8% and 26.0% rVE for aIIV3 vs. IIV4e observed for those two seasons has the potential to help further reduce the high burden of influenza among older adults [27,37]. The benefits of aIIV3 were further highlighted by reductions in IRMEs among subjects with high-risk medical conditions between 2017 and 2019, as well as reduced outpatient and inpatient IRMEs in the 2019–2020 season compared to individuals that received IIV4e and HD-IIV3. The greater reduction in inpatient admissions seen specifically with aIIV3 vs. IIV4e addresses an important public health goal to minimize the impact of influenza admissions and reduce burden on the health care, such as is observed in the COVID-19 pandemic. The advantage of aIIV3 over IIV4e was consistently observed across these health outcomes and subgroups in all three seasons. aIIV3 demonstrated improved rVE compared to HD-IIV3 in the overall age groups for prevention of IRME over the three seasons and for inpatient hospitalizations for the 2019–2020 season, but showed comparable effectiveness to HD-IIV3 in high-risk patients during two of three influenza seasons. Since HD-IIV3 likewise increases the magnitude of the immune response, the additional broadening of protection against variant strains seen with the adjuvant may account for the benefits of aIIV3 against HD-IIV3 observed in this study, but may also explain why in some analyses the rVE of the vaccines is comparable. Whether these trends will continue requires further study (i.e., of aIIV4 and HD-IIV4). Nevertheless, while the COVID-19 pandemic continues to place pressure on both outpatient and inpatient settings and drive high rates of hospitalizations, any decrease in the need for influenza-related medical care will benefit individuals, health care systems, and the public [46]. 

In sum, these findings lend support to the use of aIIV3 to reduce the burden of seasonal influenza in individuals 65 years of age or older.

## Figures and Tables

**Table 1:** Numbers of vaccinated subjects ≥65 years of age used in the analysis for each season.

Vaccine Group	2017–2018 *n* (%)	2018–2019 *n* (%)	2019–2020 * n (%)
Overall			
aIIV3	524,223 (10.9)	1,031,145 (17.9)	936,508 (27.5)
HD-IIV3	3,377,860 (70.1)	3,809,601 (66.2)	1,813,819 (53.3)
IIV4e	917,609 (19.0)	915,380 (15.9)	651,034 (19.1)
Total	4,819,692 (100)	5,756,126 (100)	3,401,361 (100)
High risk subgroup			
aIIV3	168,125 (9.6)	328,227 (16.0)	—
HD-IIV3	1,226,916 (69.9)	1,375,525 (66.9)	—
IIV4e	360,379 (20.5)	351,260 (17.1)	—
Total	1,755,420 (100)	2,055,012 (100)	—

* Subgroup analysis in the high-risk group was not performed for this season.

## Data Availability

The datasets used in this study are privately licensed and are not available in order to maintain patient privacy.

## Supplementary Materials

The following supporting information can be downloaded at: https://www.mdpi.com/article/10.3390/vaccines10091456/s1, Table S1. List of CPT, CVX, and NDC codes used to identify influenza vaccines from the Veradigm EMR dataset; Table S2. Outcome case definitions.
