# Supplementary material for: Relative Vaccine Effectiveness of Adjuvanted Trivalent Influenza Vaccine over Three Consecutive Influenza Seasons in the United States
Source: Vaccines (Basel). 2022 Sep 2;10(9):1456. doi: 10.3390/vaccines10091456 (PMC9504704; doi:10.3390/vaccines10091456)
Supplement: Supplementary file 1 [file vaccines-10-01456-s001.zip › vaccines-1842992-supplementary.pdf]

SUPPLEMENTAL MATERIAL

Evaluating Influenza Vaccine Effectiveness Over Time: Three Years of Real World Evidence from a Large Integrated Dataset

Table S1. List of CPT, CVX, and NDC codes used to identify influenza vaccines from the Veradigm EMR dataset.

Influenza vaccine type	CPT	CVX	NDC
aIIV3	90653	168	66521-0000-01, 70461-0001-01, 70461-0002-01, 70461-0018-03, 70461-0019-03, 66521-0000-11, 70461-0001-11, 70461-0002-11, 70461-0018-04, 70461-0019-04
HD-IIV3	90662	135	49281-0389-65, 49281-0391-65, 49281-0393-65, 49281-0395-65, 49281-0397-65, 49281-0399-65, 49281-0401-65, 49281-0403-65, 49281-0405-65, 49281-0393-88, 49281-0395-88, 49281-0397-88, 49281-0399-88, 49281-0401-88, 49281-0403-88, 49281-0405-88
IIV4	90685, 90686, 90687, 90688, 90630	158, 150, 161	33332-0219-20, 33332-0316-01, 33332-0317-01, 33332-0318-01, 33332-0319-01, 33332-0416-10, 33332-0417-10, 33332-0418-10, 33332-0419-10, 33332-0219-21, 33332-0316-02, 33332-0317-02, 33332-0318-02, 33332-0319-02, 33332-0416-11, 33332-0417-11, 33332-0418-11, 33332-0419-11, 58160-0896-52, 58160-0900-52, 58160-0903-52, 58160-0905-52, 58160-0907-52, 58160-0898-52, 58160-0901-52, 58160-0896-41, 58160-0900-41, 58160-0903-41, 58160-0905-41, 58160-0907-41, 58160-0898-41, 58160-0901-41, 19515-0891-11, 19515-0894-52, 19515-0895-11, 19515-0897-11, 19515-0898-11, 19515-0901-52, 19515-0903-11, 19515-0906-52, 19515-0908-52, 19515-0896-11, 19515-0900-11, 19515-0909-52, 19515-0912-52, 19515-0891-01, 19515-0894-41, 19515-0895-01, 19515-0897-01, 19515-0898-01, 19515-0901-41, 19515-0903-01, 19515-0906-41, 19515-0908-41, 19515-0896-01, 19515-0900-01, 19515-0909-41, 19515-0912-41, 49281-0413-10, 49281-0413-50, 49281-0414-10, 49281-0414-50, 49281-0415-10, 49281-0416-10, 49281-0416-50, 49281-0417-10, 49281-0417-50, 49281-0418-10, 49281-0418-50, 49281-0419-10, 49281-0419-50, 49281-0513-25, 49281-0514-25, 49281-0516-25, 49281-0517-25, 49281-0518-25, 49281-0519-25, 49281-0621-15, 49281-0625-15, 49281-0627-15, 49281-0629-15, 49281-0631-15, 49281-0413-58, 49281-0413-88, 49281-0414-58, 49281-0414-88, 49281-0415-58, 49281-0416-58, 49281-0416-88, 49281-0417-58, 49281-0417-88, 49281-0418-58, 49281-0418-88, 49281-0419-58, 49281-0419-88, 49281-0513-00, 49281-0514-00, 49281-0516-00, 49281-0517-00, 49281-0518-00, 49281-0519-00, 49281-0621-78, 49281-0625-78, 49281-0627-78, 49281-0629-78, 49281-0631-78
IIV3	90656, 90658	141, 140	33332-0010-01, 33332-0013-01, 33332-0014-01, 33332-0015-01, 33332-0016-01, 33332-0017-01, 33332-0018-01, 33332-0110-10, 33332-0113-10, 33332-0114-10, 33332-0115-10, 33332-0116-10, 33332-0117-10, 33332-0118-10, 33332-0013-02, 33332-0014-02, 33332-0015-02, 33332-0016-02, 33332-0017-02, 33332-0018-02, 33332-0113-11, 33332-0114-11, 33332-0115-11, 33332-0116-11, 33332-0117-11, 33332-0118-11

aIIV3, adjuvanted trivalent inactivated influenza vaccine; CPT, current procedural terminology; CVX, code for vaccine administered; HD-IIV3,

high-dose nonadjuvanted trivalent inactivated influenza vaccine; IIV3, nonadjuvanted egg-derived trivalent inactivated influenza vaccine; IIV4, nonadjuvanted egg-derived quadrivalent inactivated influenza vaccine; NDC, national drug code.

Table S2. Outcome case definitions.

ICD-10-CM Codes	ICD-9-CM Codes
J09 (influenza due to certain identified influenza viruses)	—
J09.X (influenza due to identified novel influenza A viruses)	—
J09.X1 (influenza due to identified novel influenza A virus with pneumonia)	488.0 (influenza due to identified avian influenza virus)
	488.01 (influenza due to identified avian influenza virus with pneumonia)
	488.8 (influenza due to novel influenza A)<br>488.81 (influenza due novel influenza A with pneumonia)
J09.X2 (influenza due to identified novel influenza A virus with other respiratory manifestations)	488.02 (influenza due to identified avian influenza virus with other respiratory manifestations)
	488.82 (influenza due to novel influenza A with other respiratory manifestations)
J09.X3 (influenza due to identified novel influenza A virus with gastrointestinal manifestations)	488.09 (influenza due to identified avian influenza virus with other
	488.1 (influenza due to 2009 H1N1 influenza virus)
J09.X9 (influenza due to identified novel influenza A virus with other manifestations)	488.19 (influenza due to identified 2009 H1N1 influenza virus with other manifestations)
	488.89 (influenza due to novel influenza A with other manifestations)
J10 (influenza due to other identified influenza viruses)	—
J10.0 (influenza due to identified novel influenza A viruses)	—
J10.00 (influenza due to other identified influenza virus with unspecified type of pneumonia)	487.0 (influenza with pneumonia)
J10.01 (influenza due to other identified influenza virus with the same other identified influenza virus pneumonia)	487.1 (influenza with other respiratory manifestations)
	487.0 (above)

ICD-10-CM Codes	ICD-9-CM Codes
J10.08 (influenza due to other identified influenza virus with other specified pneumonia)	488.11 (influenza due to identified 2009 H1N1 influenza virus with pneumonia)
J10.1 (influenza due to other identified influenza virus with other respiratory manifestations)	487.1 (above)
	488.12 (influenza due to identified 2009 H1N1 influenza virus with other respiratory manifestations)
J10.2 (influenza due to other identified influenza virus with gastrointestinal manifestations)	487.8 (influenza with other manifestations)
J10.8 (influenza due to other identified influenza virus with other manifestations)	
J10.81 (influenza due to other identified influenza virus with other manifestations with encephalopathy)	
J10.82 (influenza due to other identified influenza virus with other manifestations with myocarditis)	
J10.83 (influenza due to other identified influenza virus with other manifestations with otitis media)	
J10.89 (influenza due to other identified influenza virus with other manifestations)	
J11 (influenza due to unidentified influenza virus)	—
J11.0 (influenza due to unidentified influenza virus with pneumonia)	—
J11.00 (influenza due to unidentified influenza virus with unspecified type of pneumonia)	487.0 (above)
J11.08 (influenza due to unidentified influenza virus with specified pneumonia)	
J11.1 (influenza due to unidentified influenza virus with other respiratory manifestations)	487.1 (above)
J11.2 (influenza due to unidentified influenza virus with gastrointestinal manifestations)	487.8 (above)
J11.8 (influenza due to unidentified influenza virus with other manifestations)	
J11.81 (influenza due to unidentified influenza virus with encephalopathy)	
J11.82 (influenza due to unidentified influenza virus with myocarditis)	
J11.83 (influenza due to unidentified influenza virus with otitis media)	
J11.89 (influenza due to unidentified influenza virus with other manifestations)	
